# Off-camera gaze decreases evaluation scores in a simulated online job interview

**DOI:** 10.1038/s41598-024-60371-5

**Published:** 2024-05-31

**Authors:** Masahiro Shinya, Noriko Yamane, Yuki Mori, Brian Teaman

**Affiliations:** 1 Graduate School of Humanities and Social Sciences, Hiroshima University, Higashi-Hiroshima, Japan; 2 Faculty of Integrated Arts and Sciences, Hiroshima University, Higashi-Hiroshima, Japan; 3 Department of International and English Interdisciplinary Studies, Osaka Jogakuin University, Osaka, Japan

**Keywords:** Human behaviour, Psychology

## Abstract

During the pandemic, digital communication became paramount. Due to the discrepancy between the placement of the camera and the screen in typical smartphones, tablets and laptops, mutual eye contact cannot be made in standard video communication. Although the positive effect of eye contact in traditional communication has been well-documented, its role in virtual contexts remains less explored. In this study, we conducted experiments to gauge the impact of gaze direction during a simulated online job interview. Twelve university students were recruited as interviewees. The interview consisted of two recording sessions where they delivered the same prepared speech: in the first session, they faced the camera, and in the second, they directed their gaze towards the screen. Based on the recorded videos, we created three stimuli: one where the interviewee’s gaze was directed at the camera (CAM), one where the interviewee’s gaze was skewed downward (SKW), and a voice-only stimulus without camera recordings (VO). Thirty-eight full-time workers participated in the study and evaluated the stimuli. The results revealed that the SKW condition garnered significantly less favorable evaluations than the CAM condition and the VO condition. Moreover, a secondary analysis indicated a potential gender bias in evaluations: the female evaluators evaluated the interviewees of SKW condition more harshly than the male evaluators did, and the difference in some evaluation criteria between the CAM and SKW conditions was larger for the female interviewees than for the male interviewees. Our findings emphasize the significance of gaze direction and potential gender biases in online interactions.

## Introduction

Through 3 years of a pandemic, video calling, encompassing everything from meetings to job interviews, has become the norm. According to a report by Global Market Insights, the world's video conferencing market size was estimated at $25 billion in 2022, and it is projected to grow at approximately 10% per annum over the next decade^1^. Video calls, share many similarities with face-to-face real-life communication; however, there are still differences between them. One such difference is a lack of bidirectionality of eye contact which arises from the physical disparity in the positions of the camera and the screen. In the case of “real” communication, when one person looks at their communication partner, they can discern whether the partner is also looking back at them. In this sense, eye contact can be defined as mutual gaze. Many scientific studies have generally confirmed the positive impact of eye contact on face-to-face communication^2–4^. However, whether using a smartphone or a laptop, during online communication, if one looks at the eyes of the communication partner displayed on the screen, the partner cannot perceive eye-to-eye contact but sees the eyes as skewed (often looking down if a camera is located above the screen), and conversely, if one speaker’s gaze is toward the camera, they cannot observe the person’s face on the screen, though the other person can feel that they are being looked at directly. In a previous study, the unbalanced gaze situation encountered in video communication was termed 'skewed visuality' and was subject to qualitative investigation^5^. Previous studies have reported that people are sensitive to the gaze direction of persons in the screen and capable of perceiving whether the on-screen person’s eyes are directed at their own eyes^6^. The question we tackled in the present paper was what impact might such a gaze condition have on online communication? Or, more practically, where should we direct our gaze during online communication?

Our perception of evaluation towards others in conversation is not solely derived from the verbal content of the conversation but is influenced by eye contact and gaze behavior. According to the review of Hietanen (2018), gaze automatically provokes an observer’s positive affective responses^7^. Such affective arousal was found from both the observer and the sender of the gaze, from an experiment where no gaze, mutual gaze, and send-only or receive-only conditions were compared^8^. Benefits of eye contact not only occur with interacting dyads sharing time and space, but they also happen to individuals when the partner is not visible but they believe they are being seen by the partner, and this belief enhances self-awareness^9^. The impact of eye contact has been reported not only in interactive communication, but also in judging individuals represented in the photos. One study found that participants rated faces looking directly at them (i.e., looking at the camera) as more trustworthy and attractive^10^. De Boer et al. (2020) analyzed how viewers recognize emotions, comparing audio-only, video-only, and audiovisual conditions^11^. They found these three conditions were not different in identifying emotions. Deyne et al. (2021) concluded that to fully represent the meaning of words, multimodal information beyond pure linguistic data is necessary^12^. The importance of the non-verbal information including eye contact and gaze behavior was also confirmed in practical situations such as academic presentations^13,14^. In studies focusing on job interviews, where abstract evaluations of speakers become crucial, it was shown that candidates who displayed positive non-verbal cues like eye contact and smiling were perceived as more competent and likeable, leading to positive outcomes in the interview^15^. Conversely, candidates who displayed negative cues, like avoiding eye contact, faced diminished prospects.

Despite the efforts of many scientists, few have delved into the nuances of online interactions—particularly, the significance of a person's gaze being directed at the camera during virtual communication. In Kaiser et al. (2022), participants had a conversation with a partner about holidays for about 16 min with two conditions – one with a special camera angle manipulated so that interlocutors had eye contacts, and the other without such a manipulation^5^. After the conversation, they participated in structured interviews, regarding the overall experience, conversation itself and difference between the two conditions. Their qualitative research results indicate that interlocutors in dyads with skewed visuality sense an emotional and physical distance. In the context of medical teleconsultation, it has been reported that patients preferred camera-directed gaze and physicians with camera-directed gaze were highly rated in terms of communication skill^16,17^. Although the previous result highlighted the importance of eye contact in interactive conversation, it remains unexplored whether the skewed visuality of online communication affects the evaluation in a formal context such as online job interviews. In this context, the onus is on the interviewee not only to communicate effectively but to leave a lasting positive impression, possibly influencing a hiring decision.

The primary purpose of this study was to investigate the effect of gaze direction on an evaluation of a simulated online job interview. We hypothesized that the interviewee’s gaze toward the camera has a positive impact on the evaluation. In our experiments, we recruited twelve Japanese university students comprised of six females and six males, acting as job interview candidates. The candidates were instructed to deliver a short presentation while answering pre-set questions. The presentation was filmed in two gaze conditions: 1) candidates looking directly at the camera (CAM) that is positioned on top of the screen, and 2) candidates focusing on the eyes shown in the face image displayed on the screen, resulting in skewed visuality (SKW). Additionally, we produced a voice-only version (VO) by extracting the audio from these videos. Then, we recruited 37 full-time company employees (18 females, 19 males; age range 22–61) as evaluators. The evaluators were asked to watch the prerecorded interviewees’ presentation and to evaluate them. The results indicated that interviews in the CAM condition were rated more favorably than those in the SKW condition, confirming our initial hypothesis.

As a secondary analysis, we investigated the effect of gender of the candidates and evaluators. It has been reported that eye contact varies across genders making it an aspect of genderlect^18^. The mixed-design ANOVA revealed an interaction between gender and gaze conditions. Differences in evaluations observed between CAM and SKW conditions were more pronounced for female candidates and female evaluators, compared with their male counterparts. Although the small sample size urges us to caution in making strong claims, the results showed a potential systematic gender bias.

## Results

### Effect of interviewee’s gaze condition

**Figure 1:**
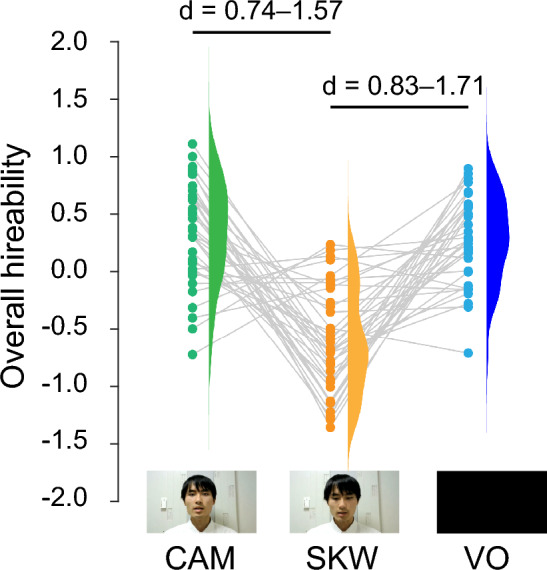
The effect of the interviewee’s gaze condition on the overall hirability. Data for each evaluator are illustrated as dots connected with gray lines. The 95% confidence intervals of the effect sizes for the significant differences revealed by the post-hoc comparisons are shown. The face of an interviewee is shown for clarity with his permission.

Significant main effects of interviewee’s gaze conditions were observed in all the outcomes we obtained (Table 1, Fig. 1). The post-hoc pairwise comparisons revealed that the evaluation scores were higher in CAM and VO conditions than in SKW condition. The differences between CAM and VO conditions were not statistically significant.

**Table 1:** Effect of interviewee’s gaze on evaluation.

Criteria	rmANOVA & Friedman tests					Effect sizes [95%CI] of paired t tests and Wilcoxon signed rank tests		
	F(2, 72)	χ^2^ (2)	p	η^2^	Kendall's W	CAM–SKW	VO–SKW	CAM–VO
Intimacy		44.811	< 0.001		0.606	0.986 [0.97–0.99]*	0.966 [0.93–0.98]*	0.238 [−0.13 to 0.55 ]
Social desirability	30.929		< 0.001	0.462		1.124 [0.71–1.53]*	0.942 [0.55–1.33]*	0.342 [0.01–0.67 ]
General job abilities	23.884		< 0.001	0.399		1.008 [0.61–1.40]*	0.917 [0.53–1.30]*	0.217 [−0.11–0.54 ]
Decisiveness	12.507		< 0.001	0.258		0.75 [0.38–1.11]*	0.594 [0.24–0.94]*	0.275 [−0.06 to 0.60 ]
Cooperativeness	21.613		< 0.001	0.375		0.971 [0.58–1.36]*	0.88 [0.50–1.26]*	0.14 [−0.19 to 0.46 ]
Overall hireability	39.033		< 0.001	0.52		1.159 [0.74–1.57]*	1.275 [0.83–1.71*]	0.085 [−0.24 to 0.41 ]

Non-parametric statistics were used for intimacy. For the post-hoc pairwise comparisons, effect sizes and their 95% confidence intervals are shown.

*p < 0.0167 (Bonferroni correction: 0.05/3).

### Secondary analysis: effect of gender

As a secondary analysis to understand gender differences, we performed three-way rmANOVAs with within-subjects effects of condition and interviewee gender, and between-subjects effect of evaluator gender. The results suggest that the difference in intimacy between was affected by the interviewees’ and evaluators’ gender: the difference between the CAM and SKW conditions were larger for female interviewees and evaluators.

**Figure 2:**
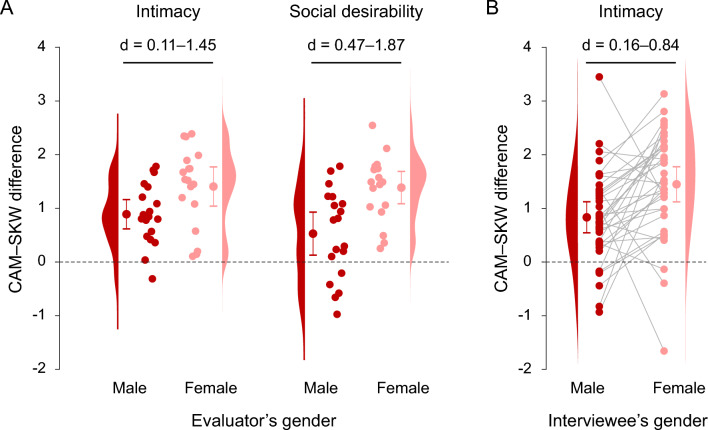
The effect of gender on the differences in evaluation scores between conditions where the interviewee’s eyes were on-camera (CAM) and off-camera (SKW). (**A**) Independent samples t tests revealed larger differences in intimacy and social desirability in the female evaluators than in the male evaluators. (**B**) A paired samples t test revealed that the difference in intimacy between the CAM and SKW conditions was larger for the female interviewees than for the male interviewees. For all the panels, figure format is the rain cloud plot where individual data points, the mean and standard deviation, and the violin plot are shown. For the (**B**), the dots connected with a grey line show the paired data associated with the same evaluator.

As summarized in Table 2, significant interactions between condition and evaluator gender were observed for intimacy and social desirability. A significant interaction between condition and interviewee gender was observed for intimacy. Other interactions were not significant. The main effect of interviewee gender and that of evaluator gender were not significant. Independent t tests revealed that the CAM–SKW differences in intimacy and social desirability were larger in the female evaluators than in the male evaluators (Fig. 2A). A paired t test revealed that the CAM–SKW differences in intimacy was larger for female interviewees than for male interviewees (Fig. 2B).

**Table 2:** Interactions between condition and genders.

	Intimacy			Social desirebility			General job abilities		
	F	p	η^2^	F	p	η^2^	F	p	η^2^
Condition × evaluator gender	5.397	0.007*	0.031	4.744	0.012*	0.038	1.988	0.145	0.018
Condition × interviewee gender	4.927	0.01*	0.027	0.246	0.782	0.002	0.195	0.823	0.001
Condition × evaluator gender × interviewee gender	0.637	0.532	0.003	1.020	0.366	0.006	0.971	0.384	0.006

	Decisiveness			Cooperativeness			Hireability		
	F	p	η^2^	F	p	η^2^	F	p	η^2^
Condition × evaluator gender	1.991	0.144	0.021	1.700	0.19	0.017	0.917	0.405	0.007
Condition × interviewee gender	0.052	0.949	0.000	1.447	0.242	0.010	0.918	0.404	0.007
Condition × evaluator gender × interviewee gender	0.147	0.864	0.001	1.753	0.181	0.012	0.391	0.678	0.003

Degrees of freedom for the F values are F(2, 70).

*p < 0.05.

## Discussion

The primary objective of this study was to investigate the impact of a candidate's gaze direction during online job interviews on their evaluation. Consistent with our hypothesis, it was demonstrated that when a candidate's gaze was directed towards the camera, they received higher ratings on all evaluation criteria compared to when their gaze was directed towards the screen (i.e., from the evaluator's perspective, the candidate's gaze was deviating downwards). In addition, we examined whether facial information from a camera-directed or screen-directed gaze positively or negatively influences in the context of an online job interview, compared to a condition where evaluation was solely based on auditory information. Our experimental results revealed that facial information from a skewed gaze negatively affected the evaluation compared to the audio-only condition, while no effect of enhancing the evaluation was observed for facial information from a camera-directed gaze compared to the audio-only condition.

Previous research on auditorily and visually conditioned research has demonstrated the combined audiovisual signals are integrated in the comprehension of the speech contents. Compared to unimodal stimuli, the multimodal stimuli such as pairs of speech and gestures provide an audience with more information that helps them to identify speakers’ emotions^11^ helps children learn^19^, facilitate presentations and discussions among adults^12,13^, and aids them in understanding the complex contents of speech^20^. In contrast to the previously reported positive impact of audiovisual integration, our results demonstrated a negative effect: the CAM condition received no better evaluation than in the VO condition. This might sound like a surprising result, but we understand that such a difference is attributed to what evaluators are searching for. In de Boer et al.’s research they evaluated the emotions of the individuals presented, but in our study judgements were made based on the thoughts and experiences of the individuals presented, which is crucial for hiring decision in this job interview context^11^. The evaluators of our study are all adults who have full-time work experience, so the language that interviewees produced seem to have been relatively comprehensible and expected even without any visual stimuli.

Multimodal signals elicited a negative evaluation when speakers gazes were skewed (SKW). One possible interpretation of the observed lower evaluation in SKW condition might be related to the negative emotion that the aversion itself might bring to the evaluators. Wirth et al. (2010) found that averted gaze leads to feelings of ostracism and negative relational evaluations in social interaction^21^. In their experiment, participants viewed a simulated 2.5-min movie made up of still images with either direct gaze, or averted gaze (with eyes oriented toward the left and right) conditions, then were instructed to visualize being in an interaction with the person represented in the movie (what they call an avatar). Averted gaze conditions led to lower perceptions of warmth and competence, resulting in feelings of being excluded and ignored. These feelings are in contrast to feelings of ‘intimacy’ and ‘social desirability’ evaluators rated low in our current experiment. Atypical eye contact during verbal communication is also known to be observed in clinical populations such as those with autism spectrum disorder (ASD), who have difficulty in social interactions with other people^22,23^. Less synchronous behavior in facial and vocal expressions is also found in ASD adolescents and children^23^. They are less coordinated with the facial and vocal expressions of others than their normally developed peers–particularly when using emotional speech. These findings highlight the crucial role of gaze in social interactions and its significant impact on how people feel about others in online contexts, imagining how they would interact with the person in real life, and perceive and evaluate their relationships with others.

The current experimental stimuli show a real person in video form while talking rather than movies constructed of non-speaking stills. In the context of a job interview, positive verbal contents and positive non-verbal behaviors are both expected, but the continuous gaze aversion in SKW condition potentially contradicts the linguistic contents of their presentation. To test the impression of receiving mismatched speech-gesture pairings, mismatch experiments were conducted with listeners^24,25^. The result reports that listeners attend to the gesture more in mismatched pairing of speech gestures, or attempt to reconcile conflicting information from speech and gesture, suggesting that gestures have a strong influence on the audience’s comprehension of the message. This result is consistent with examples of the McGurk effect which has shown that mismatches between gesture and speech confuse the understanding of spoken syllables^24,26,27^, and are perceived as deceptive^28^. People unconsciously execute and integrate multimodal information: When they ask questions, eyebrows may be raised; when they emphasize an important word in a message, the word may come with beat gestures. Likewise, when they hesitate to talk or make a pause, they may avert gaze from the interlocutor, to signal that it is their turn to talk^29^.

According to Kendrick and Holler (2017), in natural conversation, gaze aversion is more likely to occur with ‘dispreferred responses’ rather than with preferred responses^30^. This was confirmed by their eye-tracking experiment of conversation in triads. The data suggests that most dispreferred type-conforming tokens (e.g., I don’t know, dunno) occur with gaze aversion (68.2%, n = 15), in contrast to only 17.5% (n = 17) of preferred ones^31^. No data for a similar experiment is reported for the Japanese language, but it may be more natural to assume that gaze aversion may have invoked a negative impression even though the verbal information was positive. Gaze is a multidimensional feature that contributes to the impression of communication competence, helps stuttered speech to sound smooth, and makes an audience feel less distracted^32^. Overall, our experiment provided empirical evidence that online gaze aversion led to a negative impact on the evaluation in the simulated job interview session.

As a secondary analysis, we investigated the effect of gender and found that the value of eye contact was higher for females than males. The effect was observed both for evaluators and interviewees: female interviewees tended to be rated highly when they looked at the camera, and female evaluators tended to give a higher score to candidates who looked at the camera. The gender difference in communication has been reported in previous studies. For example, Tannen reported that females tend to be more engaged in gazing at human faces while talking^33^. Other researchers have shown that gaze sensitivity is higher in female than male in adults^34^, likewise for 6-month-old infants^35^, and across nationalities^36^. Coutrot et al. (2016) analyzed eye tracking data of 405 participants from 58 nationalities watching videos of another person, they found that female gazers use a more exploratory scanning strategy than males, and females watching females have a stronger left-eye bias^36^. Although the observed effect of gender may possibly be attributed to a biological difference^37^, given the insufficient statistical power in the secondary analysis of our study, we should note that future work is needed to make stronger claims about the gender-specific effect of gaze in online communication.

The effects of gender were observed in intimacy and social desirability but not in hireability, which should be considered the most critical items in the context of job interview. Although empirical data were limited for discussing potential mechanisms, it can be speculated that the results might reflect societal expectations of gender-specific communication styles. A previous paper reported that females were penalized in sociability and likeability scores if they violated stereotypical gender-inappropriate communication styles, whereas overall impression and hireability were not affected^38^. In that study, males were penalized in hireability scores for communicating in a stereotypically gender‐inappropriate manner. In the present study, the skewed gaze and the lack of eye contact by the female interviewee might be perceived as a violation of the stereotypical women's communication style, and the subscores related to stereotypical women's traits (i.e., intimacy and social desirability) might be more influenced by the gaze condition in females than in males. However, the professional evaluators who volunteered in our experiment might be aware that 'social desirability' could be influenced by eye contact but is not the major trait required in their profession. This aligns with the criticism by Griffith & Peterson (2008) that social desirability could be an unreliable measure that can be influenced by faking acts of candidates^39^. Taking the issue into account, they might have been trained in their professional career to make hiring decisions independently of potential gender bias.

The observed effect of gaze might be dependent on the experimental setting in terms of the communication context (job interview), language and culture (Japanese). Kita’s comprehensive review of cross-cultural differences in speech-accompanying gestures states that there are universal features of speech-accompanying gestures (e.g., iconic gestures to depict actions and objects), but there are also significant cross-cultural differences in the types of gestures used (e.g., politeness) and their frequency^40^. It is known that people in Asian cultures tend to avert gaze when they are thinking to answer questions^41,42^, or to interpret faces making eye contact as angrier, more unapproachable and more unpleasant than participants from a Western European country^18^. In spite of apparent East Asian listeners’ low expectations regarding gaze, our experimental results suggest that evaluators evaluate talker’s gaze aversion seriously as shown in our results. Evaluators’ written comments, which we collected during the rating task by telling them to write down if they noticed something, support these results. For example, one said: the candidate who does not look at the camera does not appeal to me. So, apparently, the lower importance of gaze is not monolithic for all cultures and contexts. In the context of a job interview, interviewers might expect the interviewees to show enthusiasm for business by directing their gaze toward the listener. Furthermore, in the result of Haensel et al. (2022)’s experiment using a storytelling game, East Asian dyads showed longer mutual gaze compared to Western Caucasians^43^. Another study reported that Chinese children’s sharing behavior increased when communicated with gaze, which is significantly larger than Americans^44^. These pieces of evidence suggest that the importance and influence of eye contact could vary across cultures, languages, and contexts.

Our experimental findings suggest several practical implications since interviewer's perceptions may be biased by the interviewee's gaze. When the interviewee's gaze is not directed towards the camera, it may not necessarily reflect an intention not to maintain eye contact, a condition that is different from a “real” conversation. In other words, due to the physical misalignment between the camera and the screen, attempting to make eye contact with the person depicted on the screen (which would presumably indicate an eagerness to communicate) could potentially give a negative impression, as it might appear to the other person that the gaze is being averted. As advice for interviewees, being aware of such constraints and intentionally looking in the direction of the camera while nodding or smiling might facilitate communication and even improve their chances of having a successful interview. From the interviewer's (i.e., the company's) perspective, even if they intend to evaluate based on verbal content, unintended bias could be introduced into the evaluation. Therefore, it is important to understand the limitation of online communication and assessments should be based on the appropriate combination of written content in online and face-to-face interviews. When we are looking to the future of virtual communication, the development of hardware and software that can enable symmetric eye contact video conferencing tools and metaverse spaces (where one is aware of being looked at by persons who are looking at her/him) may contribute to making online communication more consistent with so-called natural contexts.

Some methodological limitations should be noted. We instructed the participants to behave as they would in an actual job interview in a laboratory-based experiment, yet both the interviewees and the evaluators knew that the task they were undertaking did not directly relate to real-world benefits. In introspective reports taken from the evaluators after the experiment, which was responses to the question whether they were conscious of anything during the experiment, no evaluator explicitly stated that they were aware of the experiment's intention or hypothesis (namely, the effects of the interviewees’ gaze direction). However, we cannot deny that they might have been unconsciously aware of the independent variables in the study. Furthermore, while the gender differences suggested in the secondary analysis are intriguing, it should be noted that one must be careful when discussing the result because the findings were derived from the limited number of data and the specific experimental settings. To gain more practical insights, a sociological survey analyzing the big data of actual recruitment activities across multiple companies would be necessary.

In conclusion, our lab experiments simulating job interviews demonstrated that the direction of the interviewee's gaze significantly impacts how they are evaluated. The situation where the interviewee's gaze deviates from the camera direction by looking at the screen had a marked negative impact on the evaluation. Conversely, no significant difference was observed between the evaluations based solely on the extracted audio stimuli and those where the gaze was directed towards the camera. In addition, our secondary analysis suggested that this effect of gaze direction may vary depending on the gender of both the interviewee and the evaluator. It may be advisable for both interviewees and evaluators to be aware of such constraints and biases of online communication.

## Methods

### Experimental design

The primary purpose of this study was to examine the effect of gaze on the evaluation of an interviewee in a simulated job-interview. The statistical model for the primary purpose was a repeated measures ANOVA and post-hoc pairwise t-tests with the Bonferroni correction. We conducted a priori power analysis based on the post-hoc t-tests by using G*Power (ver. 3.1.9.7) to calculate the required sample size. The parameters for the power analysis were as follows: two-tailed paired t-test, α = 0.0167 (0.05/3), 1 − β = 0.8. A large effect size of d = 0.8 was expected according to the Cohen’s (1988) recommendation^45^. The suggested sample size was 20. The study was planned in accordance with the Declaration of Helsinki and approved by the local ethics committee at the Graduate School of Humanities and Social Sciences, Hiroshima University (approval number: HS-HUM-000523).

### Participants

**Figure 3:**
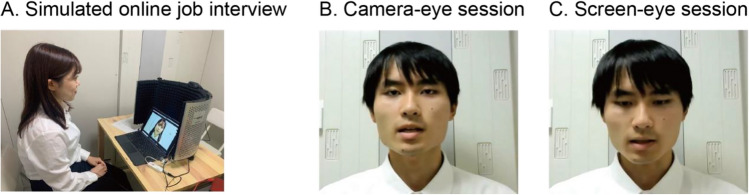
A simulated online job interview was recorded as stimuli (**A**). Two recording sessions were performed under the instruction where an interviewee was asked to look at the camera (**B**) or at the screen (where they looked at an image of themselves) (**C**). The faces of the interviewees are shown with their permission.

All the participants provided informed consent before participation. Informed consent was obtained from the participants (two participants as of Fig. 3) for publication of images in an online open-access publication. We recruited 38 Japanese full-time workers (18 females and 20 males) as evaluators. The mean age was 29.3 years (range: 22–61 years). The evaluators’ occupations varied: sales (35.1%), planning (27%), human resources (24.3%), technical professionals (13.5%), clerical staff (8.1%), school staff (8.1%), Others (8.1%). We recruited 12 Japanese university students (6 females and 6 males, 11 seniors and 1 junior) as interviewees.

### Stimuli

Simulated online job interview sessions were recorded as stimuli to present to the evaluators in a laboratory recording room where the interviewee was situated in front of a desk with a computer screen. A web camera was set on the top of the screen, approximately at eye level. We used Zoom as an online interview application. A single Japanese female played the role of interviewer for all the interviewees in the recording session. The interviewer was positioned outside of the recording room. The interview was semi-structured and the interviewer read a prepared script.

The interviewee's voice was recorded using a lavalier clip-on microphone (SONY, ECM-C115) attached to their chest, while the video was captured by the web camera on the top of the screen (720p/30fps, Logicool, C270n) and saved using the Zoom recording function. The webcam angle was adjusted for each interviewee to ensure consistent capture of their head and shoulder on the computer screen (as shown in Fig. 3A). Due to the discrepancy between the camera's position and the screen, if an interviewee looks at the screen, a skewed gaze would be captured, as illustrated in Fig. 3C. For a typical participant, with a height of 156 cm seated on a chair 48 cm high, the gaze angle during the camera-eye session was 4.8 degrees below the horizontal line, while in the screen-eye session, it was 25.6 degrees. It should be noted that these dimensions were not strictly controlled and could vary depending on the participant's posture, as we did not employ a chin rest or other equipment that could compromise the natural setting of a job interview situation. The recording room had soundproofing to prevent external noise interference. Likewise, a reflection filter was placed behind the computer to minimize any potential voice reflection against the wall.

Prior to the interview, the interviewees received advanced notification of the interview questions and the interview structure. They were instructed to prepare for the interview by formulating their responses to two questions which they were expected to answer within a time frame of 60–90 s. The interview questions were structured around i) the academic areas or extracurricular activities in which the participants invested effort during their school days, and ii) self-introduction. The participants were advised to draw from their previous or intended job-hunting experiences while responding to the questions.

The interview was conducted in Japanese (which we have translated into English). The Following is an excerpt of the interviewer’s script:

*Interviewer*: Okay, we are going to start the interview. First, please tell us about the things you put a lot of effort into when you were a student.

*Interviewee*: …

Experimenter: Thank you very much. Please continue to tell us a bit more about yourself.

*Interviewee*: …

Following a few practice sessions, two interview scenes were recorded. In one session, the interviewees were instructed to look at the computer’s camera, and in another session, to look at the computer screen which is on and their video-recorded "live" face was shown there. Note that the interviewer was intentionally not shown on the screen so that interviewees could focus solely on themselves. If realistic communication occurred between the interviewer and the interviewees, the gaze condition of the interviewees could have an unconscious influence on the interviewer's voice, attitude, and facial expressions. To eliminate such confounding factors and create a controlled stimulus, we used a semi-structured interview style during the recording of the interview, in which the interviewer read from a prepared script. In addition, the screen in front of the interviewer was turned off.

The content of both interviews needed to be the same except for possible minor variations in the wording and expressions used. Researchers confirmed that interview contents were consistent between conditions. No additional questions were asked in response to the answers provided by the participants. The interviewees were explicitly instructed not to alter their facial expressions or engage in any gestural activity during the interview sessions to minimize possible confounding effects of nonverbal behaviors other than the gaze direction. Each interview session lasted between 1 and 2 min.

The interview videos and voices were edited using Adobe Audition 2023 to normalize the maximum peak of the interviewee's voice to − 3 dB. This was done to ensure that the volume of their voice did not affect the perception of the reviewers. Additionally, audio files were extracted from the videos to create voice-only stimuli. Half of these stimuli were extracted from the camera-eye videos (Fig. 3B) and the other half from the screen-eye videos (Fig. 3C). This was done to offset any potential effects of the direction of gaze on the voice. In total, we created 36 stimuli, consisting of three conditions (CAM: camera-eye video with voice, SKW: screen-eye video with voice, and VO: voice only) from each of the 12 interviewees (6 females and 6 males). Three sets of stimuli were made to be presented to evaluators. Each stimuli set included all of the 12 interviewees and the conditions for each interviewee was counterbalanced between the stimuli sets (i.e., four CAM, four SCR, four VO conditions of different interviewees). Then we randomized the order of interviewees using a Latin square. Finally, there were 24 sequences of stimuli, one of those was assigned to each evaluator.

### Experiment: evaluating the simulated-job interview

Before evaluation, the evaluators were told to imagine they are a manager conducting the first round of interviews using Zoom, and that their task is to view videos of 1–2 min long for each interviewee only once, and to evaluate interviewees based on the given rubic. The evaluators were instructed to evaluate the interviewees' general human qualities, rather than their professional experience and skills, as this was the first screening of the interviewees.

They were instructed that the evaluation should be made in a quiet place and to wear earphones or a headset. The stimuli was delivered to the evaluators via a confidential Youtube link and the results of the evaluation were submitted via a Google form.

Six evaluation criteria were presented in the rubric. The most crucial criterion was hireability: to what extent would you want to hire the interviewee. Although there was a debate over which criteria should be considered in job interviews, we used the following five criteria based on a previous study of Wada and Wakabayashi^46^: intimacy, social desirability, general job abilities, decisiveness, and cooperativeness. According to their study, a seven-point Likert scale was used for intimacy, social desirability, and general job abilities, a five-point Likert scale was used for decisiveness and cooperativeness, and a six-point Likert scale was used for hireability. They proposed that intimacy and social desirability predict interpersonal impressions, based on their belief that personality recognition is formed from multiple factors. They employed a universal semantic differential approach to identify the potency, activity, and evaluation dimensions. Intimacy falls under the potency dimension, which represents the level of emotional comfort or familiarity with others. Social desirability falls under the activity dimension, which represents moral goodness or intellectual ability. General job abilities are under the evaluative dimension, where potency and activity dimensions intertwine. According to their factor analysis, job abilities are crucially predicted by decisiveness and cooperativeness. Hence, we adopted these five criteria in our study, and evaluators submitted six evaluation scores for each of the 12 interviewees.

### Data analysis

In order to standardize the random effects of evaluator and interviewee, the outcomes were first normalized for evaluators and then normalized for interviewees so that they have the mean value of 0 and standard deviation of 1. As the primary purpose of this study was to investigate the effect of gaze condition, the evaluation scores for 4 interviewees of the same condition were averaged. We checked the normality of the normalized variables using Shapiro–Wilk tests. Among 18 variables (3 conditions × 6 variables), no violation of normality was observed except for SKW condition of intimacy. Hence, we used a nonparametric Friedman test and a Conover test for intimacy and one-way repeated measures analysis of variance (rmANOVA) and paired t tests with Bonferroni correction for other five variables to compare the outcomes between three conditions (i.e., CAM, SKW, and VO). Before conducting rmANOVAs, the assumption of sphericity was checked using Mauchly’s test, and if the violation was observed, Greenhouse–Geisser correction was applied to the degrees of freedom for ANOVA. Significance level was set to 0.05 for the ANOVA, and 0.0167 for the post-hoc comparisons. We reported Cohen’s d as an effect size for parametric pairwise comparisons and rank biserial correlation as that for nonparametric comparisons.

The primary analysis revealed the difference between CAM and SKW conditions in evaluation scores. Then, we conducted a secondary analysis to investigate the effect of gender on the difference in the gaze conditions. For the secondary analysis, the evaluation scores for two interviewees of the same gender and condition were averaged and three-way mixed effect ANOVAs were performed. The gaze condition and interviewee’s gender were regarded as within-subject effects, and evaluator’s gender was regarded as a between-subject effect. Once significant gender-related interactions were observed, the difference between the CAM and SKW conditions were compared between genders. Independent t tests were used to test the difference between evaluator’s genders and a paired t test was used to test the difference between the interviewee’s genders. We focused on the CAM-SKW difference instead of testing all the possible pairs included in the three-way ANOVA because it was the purpose of the secondary analysis and because it was hard to adjust significance level to avoid the multiple comparison problem. We reported Cohen’s d and its confidence interval as effect sizes of the post-hoc analysis, therefore did not report p values.

## Data Availability

The data are available on request. The author MS is responsible for data requests.
